# Do fermented herbal extracts affect pig behavior, health and productivity? An on-farm study

**DOI:** 10.3389/fvets.2026.1812716

**Published:** 2026-06-04

**Authors:** Natalia Nöllenburg, Barbara Metzler-Zebeli, Katharina Schobersberger, Christoph Winckler, Christine Leeb

**Affiliations:** 1 Department of Agricultural Sciences, Institute of Livestock Sciences, BOKU University, Vienna, Austria; 2 Unit Nutritional Physiology and Functional Plant Compounds, Clinical Department for Farm Animals and Food System Transformation, University of Veterinary Medicine Vienna, Vienna, Austria

**Keywords:** animal welfare, fattening, rearing, respiratory problems, tail length

## Abstract

Respiratory disorders and tail biting represent common issues in pig husbandry. Dietary supplementation with a combination of microbes and herbal extracts could offer welfare benefits. The aim of this study was to investigate the effect of a fermented herbal extract (FHE), a blend of fermented herbs, lactobacilli and yeasts, on behavior, aspects of health and productivity in rearing and fattening pigs. Two commercial Austrian welfare label farms, raising pigs with intact tails, were included over three batches covering all seasons. Animals were kept from weaning to slaughter in control (CON; *n* = 11 and 9) and treatment pens (FHE; *n* = 11 and 9) during rearing and fattening, respectively. Behavior as well as body, tail and ear lesions were assessed at the end of the rearing and at the middle and end of the fattening period, productivity data was measured on farm while slaughter data were additionally recorded at the abattoir. Data were analyzed using (generalized) linear mixed models. One main finding of this study was the significantly lower prevalence of the indicator “tail shorter” of FHE-fed pigs compared to control (CON) pigs at the end of rearing (FHE = 7.2 %, CON = 38.8 %; *p* = 0.012). The higher prevalence of intact tails in the FHE group may suggest a beneficial effect of the supplement on behavior, with a possible reduction in tail-biting activity in rearing pigs. During fattening, FHE pigs also coughed and sneezed less during behavioral observations on farm (*p* = 0.026). Nevertheless, slaughter findings concerning the respiratory tract did not differ. Overall, the present results suggest a potential of FHE to avert abnormal behavior and promote aspects of respiratory health in rearing and fattening pigs, which warrants further investigation.

## 1 Introduction

Respiratory diseases, weaning diarrhea and tail biting are the most common challenges in rearing and fattening pigs (1–3). They are associated with reduced welfare (4), increased antibiotic use (5) and impaired productivity (6). Such challenges are usually of multifactorial origin. While tail-biting can be caused by a combination of housing (e.g., high stocking density, barren environment, and temperature changes), feeding (e.g., insufficient nutrient and water availability, inadequate feeder space ratio), as well as internal factors (e.g., impaired health status, genetic predisposition) (7), infectious diseases, specifically diarrhea and respiratory disorders, stem from combinations of various pathogens and stressors (e.g., weaning, poor air quality, and poor hygiene) and a low immune status of the herd (8, 9). Various studies point at a causal bidirectional link between tail-biting behavior and health status of pigs (10, 11) whereby poor health condition can favor tail-biting and tail-biting can induce poor health condition, for example, respiratory, enteric or locomotory problems (10). Therefore, multi-dimensional approaches are needed to possibly tackle the above-mentioned problems. While good health and the expression of normal behavior are strongly influenced by the chosen husbandry system and management practices, an additional leverage point to influence both could be the modulation of the gut microbiome and therefore the microbiota-gut-brain axis (MGBA) (12).

Probiotics, for example, members of the family *Lactobacillaceae*, which belong to the core mammalian gut microbiome (13, 14), can influence the MGBA and have attracted attention due to their capacity to interact with host neurophysiology (15, 16). Furthermore, lactobacilli are known to reduce the permeability of the intestinal mucosa, making it less susceptible to pathogens (17). They also produce short-chain fatty acids which are important for a resilient immune system and may also influence emotion and behavior (18, 19).

Next to probiotics, phytobiotics have also been used and studied in rearing and fattening pigs (20). Phytobiotics comprise herbs as well as plant-derived compounds such as essential oils, tannins or polyphenols, which have anti-microbial, anti-oxidative and anti-inflammatory properties (20, 21) and have been shown to effectively prevent weaning diarrhea (22, 23). Their application ranges from adding essential oils to mixing dried herbal blends into the regular diet. Herbal blends and extracts can influence the composition of gut microbiota. A recent study demonstrated their antimicrobial properties and stimulating effect on digestion processes (20). Phytobiotics also inhibit colonizing and spreading of harmful bacteria in the gut such as enterotoxigenic *Escherichia coli* and were even found to increase certain productivity parameters like average daily weight gain or nutrient digestibility (20, 24). While previous studies were conducted within an experimental setting, there is a lack of studies under real-farm conditions, especially using a multi-farm approach.

The goal of this study was to test the effect of a fermented herbal extract (FHE) on behavioral and health parameters in rearing and finishing pigs within a multi-farm experiment. Additionally, we investigated whether the FHE influences parameters related to growth performance, carcass characteristics and meat quality. We hypothesized that by administering FHE health issues such as respiratory disease, diarrhea and productivity could be improved and damaging behaviors like tail-biting—resulting in tail injuries and tail tissue loss—reduced.

## 2 Animals, material and methods

### 2.1 Study design

This study was initially conducted on three pig farms (A, B, C) in Upper Austria which produce for a regional Austrian meat company and welfare label (https://www.hofkultur.at/). Welfare label farms were deliberately selected because of their production standards, which address many of the known risk factors for tail-biting. Fattening pigs are kept in housing systems with twice as much space as legal minimum requirements (e.g., 1.4 m^2^/pig, 90–110 kg), a straw bedded lying area and access to an outdoor area. The farms were breeding-to-finishing pig producers, working in a three-week batch farrowing system. On all farms, farrowing sows were kept in conventional farrowing crates. Within the 1st week, all piglets received iron injections and were vaccinated routinely against *Mycoplasma hyopneumonia* as well as circo virus type 2 and were castrated under anesthesia. Due to the welfare label standards, no tail-docking was performed. All pigs were Austrian hybrids [crossbreed between Landrace × Large White (sow) and Pietrain (boar)], on average weaned at 28 days of age and slaughtered when 6–7 months old (mean: 191 days).

The study was carried out between June 2022 and July 2023 with three batches per farm in total. Each batch covered two different seasons to cover all temperature ranges. All pigs were visited twice during rearing and twice during the fattening period and followed from weaning to slaughter. Housing systems were similar (Table 1) with all study pens containing mixed groups of equal numbers of females and castrated males. All pigs were fed a standardized GMO-free diet from regional production (grains mostly produced on farm and regional protein sources; see Supplementary Table S1). Farmers were asked to provide the same conditions for all pens, for example, the same type of manipulable materials consisting usually of chains, wooden blocks, hay or straw (especially in the fattening phase) and to avoid and record all changes regarding management and feeding during the project.

**Table 1:** Description of farms (A and B) and experimental pens (control: CON and fermented herbal extract: FHE).

Parameter	Production stage	Farm A	Farm B
Farm characteristics		Farrow-to-finish farm 65 sows 450 fattening places	Farrow-to-finish farm 100 sows 400 fattening places
Feeding type		Spotmix system, dry	Spotmix system, dry
Animal: feeder place ratio	Rearing	1:1	1:4
	Fattening	1:3	1:2
Space allowance	Rearing	0.9 m^2^/piglet	0.3 m^2^/piglet
	Fattening	1.5 m^2^/pig	1.2 m^2^/pig
Husbandry system	Rearing	Outdoor climate barn with three functional areas: lying: covered area with straw bedding and heating Feeding: feeding troughs, deep straw bedding Defecation/drinking: slatted floor	Partially slatted floor, no functional areas
	Fattening	Indoors fully slatted floor Outdoor run with straw bedding	Indoors straw bedding Outdoors partially slatted floor
Number of pens/batch (CON/FHE)	Rearing	2/2 (= 4) last batch: 1/1 (= 2)	2/2 (= 4)
	Fattening	1/1 (= 2)	2/2 (= 4)
Total number of pens (CON/FHE)	Rearing	5/5	6/6
	Fattening	3/3	6/6
Pigs per pen (CON/FHE)	Rearing	22/22	20/20
	Fattening	45/45	20/20

Due to complications in correctly administering FHE to rearing and finishing pigs and time constraints, which did not allow to repeat batches, farm C needed to be excluded from the final dataset. It is nevertheless mentioned in this paper to indicate potential challenges when using FHE. In farm C, the liquid FHE was inadequately applied to the feeding system. When it was mixed with dry feed in a small feed auger, feed particles adhered to its surface and were not removed frequently enough. This created favorable conditions for mold growth. As a learning outcome of this study, we recommend all farmers to regularly check their feed augers when using FHE.

During the rearing period, on both farms (A & B) the studied animals were housed in two treatment (FHE) and two control (CON) pens each, which were located next to or in front of each other in the same compartment. The treatment group was fed a feed mixture which contained a liquid fermented herbal extract (FHE—see chapter 2.2) added to each meal, whereas the CON group received the same feed mixture without FHE. Animals remained in the same treatment group during the entire experiment. At the beginning of the study, all piglets were assigned semi-randomly by the farmer to FHE or CON pens (balancing the allocation of litters and sex). Piglets stayed in the rearing pens until they reached approximately 36 kg (mean ± SD: 35.7 ± 6.9 kg). For the fattening period, all pig groups were moved to a different compartment. On farm A the two treatment and the two control groups were merged into a bigger pen each, while group composition remained unchanged on farm B. All researchers involved were blinded until the end of data collection, as only the farmers were aware of the allocation of pens to treatments.

### 2.2 Treatment

The feed supplement used was a fermented herbal extract (FHE) in liquid form which is listed for use on certified organic farms (25) and manufactured by an Austrian company (Multikraft, Pichl bei Wels, Austria). The extract is obtained by fermenting a mixture of sugar cane molasses, lactose powder, and various plant juices, namely from *Betula pendula, Rubus idaeus, Carum carvi, Achillea millefolium, Foeniculum vulgare, Thymus vulgaris, Salvia rosmarinus, Mentha* × *piperita, Althaea officinalis* root, *Pimpinella anisum, Solidago virgaurea*, and *Silybum marianum* seeds. The fermentation process involves different strains of *Lactobacillaceae* (*Lacticaseibacillus casei, Limosilactibacillus fermentum, Plantilactobacillus plantarum*, and *Lacticaseibacillus rhamnosus*) and yeast (*Saccharomyces cerevisiae*). The FHE was mixed into the usual feed mixture at a dosage of 1% FHE per kg feed (88% dry matter). During the experiment, the FHE tank was connected to the feed pre-mixing container *via* a hose. As soon as the dry feed mixture was poured into the premix container, the liquid FHE was added to the mixture. The homogeneously mixed feed (+FHE) was then moved to the respective troughs *via* the tubular track system “Spotmix” (Schauer, Agrotonic, Prambachkirchen, Austria).

### 2.3 Data collection

Farms were visited four times in total per batch by the same observer. The 1st visit took place shortly after weaning (approx. 4 weeks old), the second at the end of the rearing period (9–12 weeks old), the third around mid of fattening (18–21 weeks old) and the fourth assessment at the end of fattening, one day prior to slaughter (26–28 weeks old).

Behavioral data were not gathered during the first visit as rehousing and -grouping would have strongly influenced “normal” behavior. During visit two, three and four, behavioral observations on pen-level were conducted using a mixture of scan and direct continuous sampling (Table 2). After a 2-min period to standardize the reaction of pigs toward the observer, the number of active and resting pigs was counted as well as the tail positions of all active pigs determined. This scan sampling was repeated after min 5 (without tail positions) and again after min 10. In between, pigs were continuously observed for a total of 10 min. This was repeated once and both observations were carried out between 09:30 am and 1:30 pm.

**Table 2:** Ethogram for continuous behavioral observations and scan sampling.

Behavior	Definition
Active (27)	Sitting, standing or moving in the pen
Comfort (27)	Rubbing or scratching on barn/objects or with hindleg
Play^*^ (individual or in a group)	Pivoting (28), hopping (28), jumping, gamboling (27), scampering (29), running alone or chasing, jumping, romping (30), play-fighting in a group without injuries and screams
Manipulation (30)	Sniffing, rooting, licking, nibbling, sucking, biting, chewing
Manipulation of environment (30)	
Object or enrichment material	Manipulation of chains, caoutchouc, wooden sticks, straw
Pen equipment	Manipulation of wall, floor, feeder, drinker
Manipulation of conspecifics (with or without causing injury)	
Pen-mate (31)	Manipulation of body except tail/ear
Ear (32)	Manipulation of ear
Tail (31)	Manipulation of tail
Tail position	
Curled or erected (33)	Curled: tail forms a loop and is in an upward position Erected: tail too short to form a loop but is kept in an upward position
Hanging (still or wagging) (33)	Tail hangs loosely toward the floor or wags from side to side
Tucked (34)	Tail is directed toward the floor and tucked between the hindlegs toward the belly
Oral stereotypies (35)	
Bar biting	Animal bites the bars of the feeding trough or barn
Empty chewing	Animal chews with empty mouth with or without foam
Tongue rolling	Animal moves its tongue by rolling, twisting, sticking it out
Coughing/sneezing (35)	Loud and fast ejection of air through mouth or nose

^*^see Supplementary material.

After the behavioral observation, the prevalence of clinical parameters (Table 3) was assessed at pen-level by entering the pen and looking at each animal. At last, fecal samples of twelve selected focus animals per batch (6 FHE and 6 CON animals) were collected, but the respective results are part of a different paper and therefore reported elsewhere (26).

**Table 3:** Description of clinical parameters (prevalence in %, except signs of diarrhea: categorical).

Clinical parameter	Definition
Ear tip injury/ear bottom injury (35)	Score 1—slight: fresh blood, crust Score 2—severe: part of the ear missing
Tail injury (36)	Score 1—slight: fresh blood, crust Score 2—medium: blood/crust and signs of infection (swollen tail) and/or necrosis Score 3—severe: open wound, obvious infection, deeper tissue visible
Tail length (36)	Score 1—no tissue loss: tail can curl but no tassel Score 2—slight tissue loss: tail cannot curl anymore but tip of rolled tail reaches body Score 3—medium tissue loss: tip of the tail cannot reach the body anymore and forms a semicircle Score 4—severe tissue loss: tail cannot form any shape and only a stump or no tail is visible
Skin scratches shoulder/skin scratches flank (35)	More than 2 scratches with a length of >3–5 cm (rearing piglets) or >5 cm (fattening pigs)
Round skin lesions (shoulder and flank) (35)	Coin-sized injured area of >1 cm diameter (rearing piglets) or >2 cm (fattening pigs)
Signs of diarrhea (35)	Pen exhibits signs of diarrhea: feces are changed in color (from dark brown to cream) and texture (from firm to liquid)
Lameness (35)	Score 1—medium: resting the foot while standing or limping when moving Score 2—severe: no weight bearing on limb possible, animal unable to walk or permanently lifting lame leg
Runt (36)	Obviously smaller piglets/pigs combined with at least one of the following indicators: visible spine, pale, hairy coat, long face, large ears, and sunken flank

All pigs were weighed on pen level after weaning and at the end of rearing, before being moved to the fattening pens. The difference between the live weight at the end and the beginning of the rearing period divided by the number of days spent in the rearing unit resulted in the average daily weight gain (ADG g/day). To determine ADG for the fattening period, the difference between the final weight after slaughter and live weight at the end of rearing (=beginning of fattening) was taken and average daily weight gain calculated. The pigs' final weight was estimated based on the carcass weight [carcass weight = live weight ^*^ 0,8; (37))]. In addition, farmers were asked to record all medical treatments, animal losses and other incidences on individual level (e.g., transfer to sick pen) as well as pen level (e.g., tail biting outbreak).

Additionally, slaughter data of pigs of all batches and farms were gathered. Trained veterinarians employed at the slaughterhouse routinely recorded all pathological changes of pig organs and carcasses. The prevalence of abscesses, pneumonia, pericarditis, pleurisy and adhesion of the pluck on pen level was considered for further analysis (38). To be able to assign slaughter numbers to treatment groups and pens, pigs were color marked the day prior to slaughter, and the slaughterhouse personnel and the responsible scientist ensured that all pigs of the present study were slaughtered continuously and not mixed between pens (and color), so that carcasses could be allocated to respective pens and treatment.

### 2.4 Data processing

While behavioral data were expressed in events per 100 animals in 10 min, clinical, productivity and slaughter findings were calculated as percentage of animals affected per pen (prevalence, in %).

Data on round lesions, ear injuries, lameness, and runts were gathered for both growing stages but occurred too rarely to be statistically analyzed (median = 0 for all parameters). Post-weaning diarrhea was only observed categorically (yes/no) on pen-level and occurred only on a very low level. It was mostly observed during the first visit of the rearing period in three control and four FHE pens across all batches and farms and was only found in one FHE pen during the second visit. Due to zero inflation, the statistical model for mortality during rearing and fattening did not converge and mortality was thus not further analyzed.

Concerning behavior, play was observed only sporadically, but when observed, usually more than one pig displayed locomotory play. As play behavior occurred infrequently (median = 0 in both treatment groups and growing stages), it was not further considered.

As ear injuries were very rare, the two score categories (1 = slight, 2 = severe) were merged to ensure sufficient data for analysis. The three scores for tail injuries (1 = slight, 2 = mild, 3 = severe) were also combined into a single category. Likewise, scores two to four of “tail length” (2 = slight tissue loss, 3 = medium tissue loss, 4 = severe tissue loss) were grouped into the category “tail shorter”, as they all indicated clear tissue loss and score four was too rare to be kept separately.

In some pens the prevalence of pigs with shorter tails decreased over time. However, the prevalence of “tail shorter” is an indicator that can only increase during the life of a pig, since lost tissue cannot grow back. The observer may have allocated tails which were in between two tail length categories differently from one visit compared to another (e.g., last visit during rearing phase to first visit during fattening). Therefore, pens were excluded from the fattening phase if the prevalence of short tails at one visit was more than 10% lower than during the previous visit (e.g., 100% of pigs had shorter tails at the end of rearing, but only 48% at mid-fattening). We tolerated deviations of up to 10%, as missing two animals out of 20 may happen, whereas larger discrepancies would be unlikely. If the deviation between end of rearing and middle/end of fattening was smaller than 10%, we assumed the value at the end of rearing to be correct and replaced the mid-/end-fattening value accordingly. Therefore, some pens were excluded regarding the tail length parameter during fattening (more details in Supplementary Table 5). Consequently, for data collected during fattening, the indicator “tail shorter” is based on a different number of pens than the other indicators.

### 2.5 Statistical analysis

Behavioral, clinical, productivity data and slaughter findings were analyzed on pen level. The statistical analysis was carried out using the SAS/STAT statistical analysis software version 9.4. Generalized linear mixed models (proc glimmix) were used for behavioral (poisson distribution) and clinical indicators (beta distribution) as well as slaughter findings (poisson distribution). For the fattening phase, fixed effects contained treatment, visit and the interaction thereof. Additionally, the prevalence of short tails at the end of rearing was included as a covariate for the indicator “tail length” to account for a reduction in tail length already during the rearing phase. For the rearing phase, only visit two was included in the statistical analysis as baseline clinical parameters during visit one were scored zero for most piglets in both groups (except farm B, batch 3: FHE-−14% tail injuries; 5% tail shorter; batch 2: 28% tails injuries; 10% tail shorter).

For the rearing phase, since only the second visit was included in the final model, random effects in the behavioral data were pen nested in treatment, batch and farm, while it was batch nested in farm for clinical data. For the fattening phase, since two visits were considered for analysis, the random effect concerning the behavioral data contained the visit (nested in pen, treatment, batch, and farm), whereas for the clinical data, the pen level was included as a random effect (nested in treatment, batch, and farm).

Linear mixed models (proc mixed) were used for ADG, lean meat proportion, meat pH and carcass weight as they met the requirements regarding normal distribution and homogeneity of residuals. The only fixed effect was treatment. Random effects included the pen nested in treatment, batch and farm.

The Benjamini-Hochberg correction of significance levels was applied to control for false discovery rate (39). This was carried out for the different indicator groups, namely behavior, injuries, slaughter findings, and productivity measures for the respective production stages (rearing and fattening). The adjusted levels of significance are indicated below the respective tables.

## 3 Results

Regarding behaviors displayed, no significant differences were observed between FHE and CON animals (Table 4). During fattening, active animals were observed less frequently at the second visit.

**Table 4:** Occurrence of behaviors (number of events/100 animals/10 min; LS-means ± standard error of means) for rearing and fattening pigs in fermented herbal extract (FHE) and control groups (CON) during visit 1 and 2 (rearing pigs: FHE *n* = 10 pens; CON *n* = 10 pens; fatteners: FHE *n* = 9 pens; CON *n* = 9 pens).

Behavior	Visit 1		Visit 2		*p*-values		
	FHE	CON	FHE	CON	T	V	T^*^V
Active^1^—Rearing	-	-	43.9 ± 7.8	37.9 ± 6.8	0.566	-	-
Active^1^—Fattening	54.3 ± 7.3	51.2 ± 6.9	31.6 ± 4.3	37.1 ± 5	0.707	**0.003**	0.429
Comfort—Rearing	-	-	0.6 ± 0.4	1.4 ± 0.9	0.438	-	-
Comfort—Fattening	1.8 ± 1.5	0.5 ± 0.4	0.4 ± 0.4	0.8 ± 0.7	0.675	0.638	0.270
Manipulation object—Rearing	-	-	24.2 ± 9.1	17.8 ± 6.8	0.573	-	-
Manipulation object—Fattening	30.2 ± 7.3	24.6 ± 6	26.4 ± 6.4	22.4 ± 5.4	0.453	0.635	0.930
Manipulation pen—Rearing	-	-	8.9 ± 4	9.1 ± 4.2	0.963	-	-
Manipulation pen—Fattening	22 ± 11.5	22.4 ± 12	2.5 ± 1.4	12.9 ± 6.7	0.128	0.015	0.135
Manipulation pen-mate—Rearing	-	-	12.3 ± 4.5	13.6 ± 5	0.847	-	-
Manipulation pen-mate—Fattening	10.6 ± 3.6	14.3 ± 4.8	10.5 ± 3.5	7.6 ± 2.6	0.972	0.345	0.372
Manipulation ear—Rearing	-	-	4.3 ± 1.8	5.3 ± 2.2	0.744	-	-
Manipulation ear—Fattening	3.6 ± 1.5	4 ± 1.7	2.5 ± 1.1	1.4 ± 0.6	0.614	0.123	0.464
Manipulation tail—Rearing	-	-	2.2 ± 1.4	1.4 ± 1	0.664	-	-
Manipulation tail—Fattening	2.7 ± 1.8	1.9 ± 1.4	1 ± 0.7	0.2 ± 0.2	0.220	0.041	0.425
Oral stereotypies—Fattening	6.1 ± 2.6	6.3 ± 2.7	12.9 ± 5.4	9.6 ± 4.1	0.750	0.181	0.699
Tail curled^2^—Rearing	-	-	41.1 ± 8.9	22.3 ± 4.9	0.061	-	-
Tail curled^2^—Fattening	66.5 ± 8.5	68.1 ± 8.7	45.6 ± 5.9	53 ± 6.8	0.498	0.019	0.626
Tail hanging^2^—Rearing	-	-	18.3 ± 8	16.4 ± 7.2	0.859	-	-
Tail hanging^2^—Fattening	9.4 ± 5.5	3 ± 1.8	2.5 ± 1.5	0.5 ± 0.3	0.038	0.018	0.725

*P*-values are given for the effects of treatment (T), visit (V) and the interaction term (T^*^V); significant differences indicated in bold letters; after Benjamini-Hochberg correction the adjusted significance levels are *p* < 0.0055 for rearing and *p* < 0.005 for fattening pigs; “-”: effect not included in the model; ^1^expressed as proportion of animals during three scans (beginning, middle, and end of the 10-min observation period); ^2^expressed as proportion of animals displaying the respective tail position during two scans (beginning and end of the 10-min observation period).

**Figure 1:**
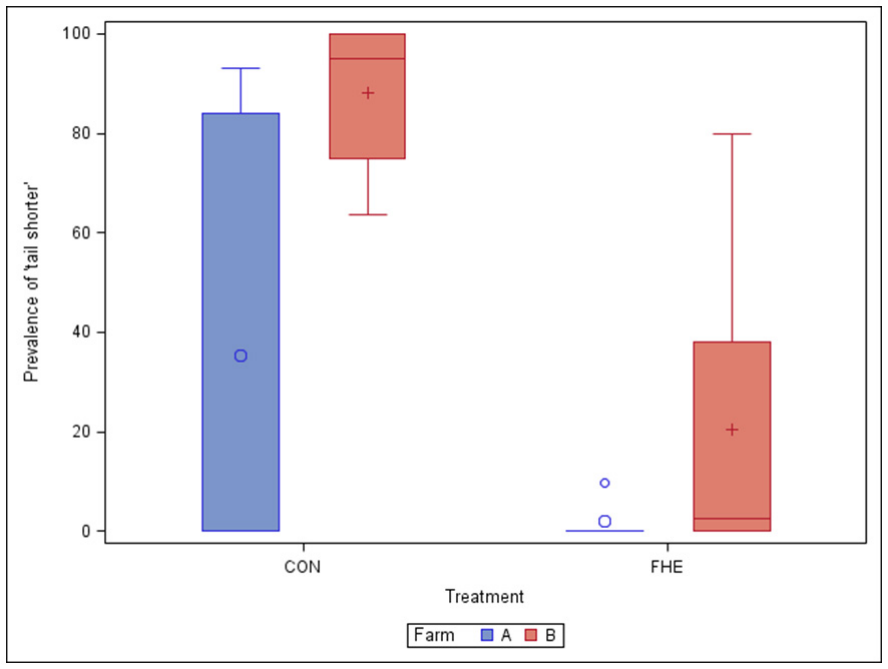
Boxplots of prevalence of “tail shorter” per farm (A and B) in the rearing phase; *p* = 0.012, after Benjamini-Hochberg correction the adjusted significance level is *p* < 0.0125 [fermented herbal extract (FHE): *n* = 11 pens; control: (CON) *n* = 11 pens] Boxplots with medians (line within the box), lower and upper interquartile range (box), whiskers representing 1.5 times the interquartile range or minimum/maximum values and the estimated means (o or +).

Results of clinical indicators are presented as an overview of injuries (Table 5) and coughing/sneezing and slaughter findings (Table 6). The prevalence of rearing pigs with shorter tails was significantly lower at the end of the rearing phase in FHE compared to CON (LS-mean FHE = 7.2 %; CON=38.8 %; p_treatment_ = 0.012; Figure 1). When accounting for tail length at the end of rearing by including it as a covariate in the statistical model for the fattening period (p_covariate_ < 0.001), no treatment effect on the prevalence of “tail shorter” was found for this phase (p_treatment_ = 0.942).

**Table 5:** Prevalence of injuries (LS-means ± standard error of means) for fermented herbal extract (FHE) and control groups (CON) during visit 1 and 2 (rearing pigs: FHE *n* = 11 pens; control *n* = 11 pens; fatteners: FHE *n* = 9 pens; control *n* = 9 pens).

Injury	Visit 1		Visit 2		*p*-values		
	FHE	CON	FHE	CON	T	V	T^*^V
Tail injury—Rearing	-	-	9 ± 5.2	8.4 ± 4.8	0.962	-	-
Tail injury—Fattening	3.6 ± 1.8	2.4 ± 1.3	5.1 ± 2.4	2.9 ± 1.6	0.475	0.397	0.838
Tail shorter—Rearing	-	-	7.2 ± 4.7	38.8 ± 12.2	**0.012**	-	-
Tail shorter—Fattening	28.8 ± 18.7	28.9 ± 18.3	28.9 ± 18.7	33.3 ± 19.9	0.942	0.347	0.364
Round lesions—Fattening	1.3 ± 0.8	0.9 ± 0.6	3.4 ± 1.8	2.9 ± 1.6	0.712	**0.007**	0.709
Scratch front—Rearing	-	-	4.6 ± 2.1	4.4 ± 2	0.882	-	-
Scratch front—Fattening	2.2 ± 1.6	0.7 ± 0.5	1 ± 0.7	1.2 ± 0.1	0.754	0.870	**0.007**
Scratch back—Rearing	-	-	5.1 ± 1.7	6 ± 1.8	0.741	-	-
Scratch back—Fattening	1.4 ± 1	0.9 ± 0.7	3.3 ± 2.3	3.2 ± 2.2	0.819	**< 0.001**	0.469

*P*-values are given for the effects of treatment (T), visit (V) and the interaction term (T^*^V); significant differences indicated in bold letters; after Benjamini-Hochberg correction the adjusted significance levels are *p* < 0.0125 for rearing and *p* < 0.01 for fattening pigs; “-”: effect not included in the model.

**Table 6:** Prevalence of coughing/sneezing and slaughter findings (LS-means ± standard error of means) for fattening pigs in fermented herbal extract (FHE) and control groups (CON) during visit 1 and 2 (rearing pigs: FHE *n* = 10 pens; control n=10 pens; fatteners: FHE *n* = 9 pens; control *n* = 9).

Coughing/sneezing and slaughter findings	Visit 1		Visit 2		*p*-values		
	FHE	CON	FHE	CON	T	V	T^*^V
Cough/sneeze^1^—Rearing	-	-	0.9 ± 0.7	1.3 ± 1	0.725	-	-
Cough/sneeze^1^—Fattening	2.5 ± 1.5	7.5 ± 4.4	0.4 ± 0.3	2.35 ± 1.4	**0.026**	**0.021**	0.562
Pneumonia—Fattening	-	-	50.1 ± 9.9	63.5 ± 12.4	0.413	-	-
Pleurisy—Fattening	-	-	4.8 ± 2.2	6.5 ± 3	0.644	-	-
Pericarditis—Fattening	-	-	1 ± 5.2	2.6 ± 1.1	0.186	-	-
Adhesion of the pluck—Fattening	-	-	0.8 ± 0.5	3.7 ± 1.7	0.069	-	-
Abscesses—Fattening	-	-	3.7 ± 1.7	2 ± 1	0.392	-	-

*P*-values are given for the effects of treatment (T), visit (V) and the interaction term (T^*^V); significant differences indicated in bold letters; for cough/sneeze a significance level of *p* < 0.05 was set; after Benjamini-Hochberg correction the adjusted significance level for all slaughter findings is *p* < 0.01; “-”: effect not included in the model, ^1^expressed as number of events/100 animals/10 min (LS-means ± standard error of means) for rearing and fattening pigs in fermented herbal extract (FHE) and control groups (CON).

Round lesions generally occurred rarely in FHE and CON groups during fattening and did not differ significantly between treatment groups. The prevalence of round lesions and scratches on the backside of the body significantly increased toward the end of the fattening phase (p_visit_ = 0.007), while a significant interaction between treatment and visit was found for scratches in the front body part with more FHE animals having scratches during the middle of fattening (LS-mean FHE = 2.2 %; CON = 0.7 %; ${\text{p}}_{\text{visi}{\text{t}}^{*}treatment}$ = 0.007).

Table 6 summarizes data on respiratory symptoms (coughing/sneezing) and slaughter findings. FHE fatteners coughed and sneezed less than controls (LS-means for number of events/100 animals/10 min: FHE = 0.4, CON = 2.35; p_treatment_ = 0.026). Independently from the treatment group, pigs coughed/sneezed significantly more during the middle of the fattening phase (p_visit_ = 0.021) compared to the end of fattening. Concerning the slaughter findings, there was no significant treatment effect.

There was no effect of FHE on mortality and productivity parameters (Table 7). Although not tested statistically, mortality was low and did not differ numerically (mean value, rearing pigs: FHE = 0.8 %, CON = 0.4 %; fatteners: FHE = 1.3 %; CON = 1.8 %).

**Table 7:** Productivity data (LS-means ± standard error of means); ADG (g/day); lean meat (%), pH and carcass weight (kg) assessed at the abattoir (rearing pigs: only ADG: FHE *n* = 11 pens; CON *n* = 11 pens; fatteners: FHE *n* = 9 pens; CON *n* = 9 pens).

Productivity data	FHE	CON	*p*-value *T*
ADG—Rearing	0.5	0.5	0.361
ADG—Fattening	0.8	0.7	0.741
Carcass weight (kg)—Fattening	95.5 ± 2.5	95.6 ± 2.5	0.984
Lean meat (%)—Fattening	61.2 ± 0.5	61.3 ± 0.5	0.817
pH of meat—Fattening	6.3 ± 0.04	6.26 ± 0.04	0.201

*P*-values are given for the effect of treatment (T); after Benjamini-Hochberg correction the adjusted significance levels are *p* < 0.05 for rearing (ADG) and *p* < 0.0125 for fattening pigs.

## 4 Discussion

Previous studies on the subject were mostly conducted on one experimental farm, providing important scientific insights. To apply and expand this knowledge, our study represents one of the few approaches investigating the effect of FHE across two farms. While we are aware of the farms' heterogeneities, especially regarding housing of rearing pigs, the approach increases external validity as it accounts for variability in real farm conditions, which may also lead to a higher acceptance of results by farmers. Furthermore, most influencing factors were comparable between the two farms and blinding the observer added to the credibility of the study.

### 4.1 Manipulative behavior, ear and tail injuries

Studies in rodents (40, 41) and humans (42, 43) have shown that feeding probiotic bacteria of the genus *Lactobacillus* can not only have beneficial health effects but also influence emotions and, consequently, behavior. Based on these findings, we tested whether such behavioral changes, specifically a reduction in redirected manipulative behavior or activity, could be observed in the treatment pens. However, behavioral indicators and activity of rearing and fattening pigs during our assessment periods did not differ significantly between treatment groups thus not confirming anecdotal farmer reports.

Pigs spend 52% of their daily activities on exploring, rooting and grazing and 23% on locomotion (44, 45). Therefore, exploration is an elementary part of their natural behavior which is a basis to ensure good welfare (46). In our study, pigs could explore—to various degrees and combinations—straw, wooden sticks and chains with/without caoutchouc toys on both farms, even though the rearing pens on farm B provided only minimum levels (wood and chain). The relatively high level of enrichment material (compared to fully conventional housing) might have overshadowed any potential treatment effect. Also, against our hypothesis, that FHE-fed pigs would show less manipulation of the pen or of pen-mates (body, ear, and tail) and less oral stereotypies, there were no significant differences between treatment groups concerning the aforementioned behaviors (during rearing and fattening phase).

The low occurrence of oral stereotypies as well as ear-, tail and body directed manipulation is also documented in similar housing systems (47, 48) and may be a hint that husbandry conditions were able to alleviate such behaviors (49) as outdoor run, straw and other organic material was provided on both farms (except during rearing on farm B). Furthermore, restrictive feeding is another factor which can promote redirected behavior and oral stereotypies but was not the case on both farms as they provided *ad libitum* feeding, controlled by sensors (50).

There was also no significant difference between FHE and CON animals concerning manipulation of the tail and tail injuries in both production stages. However, the prevalence of the indicator “tail shorter” was significantly lower in FHE pigs compared to CON at the end of the rearing phase. Tail-in-mouth behavior occurred more frequently during rearing than fattening but it is noteworthy that in the rearing system of farm A, tail-in-mouth behavior was expressed less compared to farm B, probably due to the fact that farm A provided more space, straw and an outdoor area (see Table 1). As the tail-in-mouth behavior only reflects a short-term situation, tail lesions constitute the outcome of more severe tail-biting events. In both treatment groups, the prevalence of tail injuries was relatively low (ranging from 2.9 to 13.4 %) compared to other studies (51, 52), which may have reduced the power to detect treatment effects. While injuries may heal between visits and therefore mask a potential effect of FHE, tail tissue loss remains visible and measurable as reduced tail length, so that the number of pigs with shorter tails at the end of rearing (or fattening) represents a cumulative effect of all severe tail-biting events. One main finding of this study is the significantly lower prevalence of the indicator “tail shorter” of FHE-fed pigs at the end of rearing (LS-means: FHE = 7.2 %, CON = 38.8 %; *p* = 0.012). For the finishing phase, no treatment effect was found, when the prevalence of “tail shorter at the end of rearing” was included as a covariate (p_treatment_ < 0.001) which indicates that the variation in tail length observed in the fattening period was explained by differences already present at the end of rearing. This underlines the importance of the rearing phase in determining tail outcomes later in life as also discussed by Moinard et al. (53) who argued that a more complex and enriched post-weaning environment might also reduce tail biting during later production stages. Yet, evidence-based and conclusive data on the development of tail biting, linking the post-weaning/rearing phases to the fattening phase, are lacking. Especially for pigs with undocked tails entering improved husbandry systems in the fattening phase, it is crucial to ensure appropriate husbandry conditions early in life, particularly during the suckling and weaning phases.

The differences found between treatment groups concerning tail length are particularly noteworthy in view of the farm-specific variations in rearing and fattening systems as well as in feeding technology and feed type. Even though the two selected farms are part of the same welfare label, their rearing systems were differently structured (see chapter 2, table 1): while farm B kept their animals indoors in flat decks with mostly slatted flooring, farm A had a newly built naturally ventilated open-sided barn with three functional areas and straw bedding. It is noteworthy that farm A had less pronounced and less frequent tail biting incidents compared to farm B, indicating that the husbandry system plays a crucial role in the development of redirected manipulative behavior. The fattening system was designed according to higher welfare standards on both farms, but even here there were variations: Whereas farm A kept the animals in barns with fully slatted flooring inside and deep straw bedding in the outdoor run, farm B offered minimal straw bedding (barely covering the floor) indoors and partly slatted flooring in the open-front part of the barn. Group and pen size were also smaller on farm B compared to farm A.

Tail posture was not significantly different between treatment groups. As tail postures were assessed using scan sampling during two ten-minute continuous observations, the chosen time period was most likely not representative of a pig's entire daytime activity. Moreover, tail posture changes frequently over the course of the day, but also within minutes according to their general activity.

All behavioral results represent an interesting snapshot of exploratory behavior which is directed toward materials (normal) or conspecifics (redirected) (54). Since two times 10 min per pen and visit may not reflect the pigs' overall daily activity, behavioral indicators would have benefitted from extending the observation period or even installing cameras to record entire days.

### 4.2 Scratches, social and comfort behavior

The prevalence of skin scratches, as an indicator of agonistic behavior, was on a low level and did not differ significantly between treatments. This could mean that general space allowance as well as animal to feeder place ratio were rather adequate as skin scratches can arise from competition at the feeder or for the resting area (55).

Pigs scratching or rubbing against an object were seen very rarely and no significant difference between treatment groups was found. However, interpreting this behavior is challenging as it can be comfort behavior, for example, after wallowing in order to remove dried mud (44, 56), or indicate ectoparasites (57), while it may have also been a response to the presence of an observer (58).

### 4.3 Coughing, sneezing and slaughter findings

The level of coughing/sneezing was comparable to other studies (9). Interestingly, FHE-pigs coughed and sneezed less during the fattening period compared to CON pigs (*p* = 0.026). This may indicate a beneficial effect of FHE on the respiratory tract. Such an effect could be related either to immunomodulatory properties or to direct antimicrobial effects of the fermented herbs, since the fermentation process increases the bioavailability of the ingredients (59). Additionally, for example, *Thymus vulgaris* and *Mentha* × *piperita* are commonly used in phytotherapy for respiratory tract infections (60). However, the FHE effect on coughing and sneezing was not mirrored in the slaughterhouse findings, as the prevalence of pneumonia, pleurisy and pericarditis did not differ between treatment groups. Signs of pneumonia were frequently found in both groups (LS-means: FHE = 50.1 % vs. CON = 63.5 %) and more pronounced compared to other studies (38). It is known that improved husbandry systems based on the provision of straw can have negative effects on lung health (61) as especially small dust particles (PM_10_) can carry pathogens right into the lung (62).

No significant treatment effects on diarrhea were observed, likely due to its overall very low occurrence. Moreover, as FHE supplementation started only at weaning, potential effects on gut health may not have had sufficient time to develop. Further research could focus on an earlier application of FHE within the pig production stages. Administering FHE to pregnant sows and/or to their suckling piglets can be hypothesized to have a positive impact not only on weaning diarrhea, but also on other aspects of sow and piglet health.

### 4.4 Productivity and meat quality parameters

There are several studies investigating the effects of probiotics (63–66) and phytobiotics (24), with differing outcomes. We could not find any significant differences between treatment groups concerning average daily weight gain (ADG), carcass weight, lean meat proportion or meat pH. This is in line with Davis et al. (67) who could also not find a significant difference in ADG between *Bacillus*-fed and control pigs. However, Yu et al. (68) found a higher ADG in groups receiving feed supplemented with *Lactobacillus fermentum* compared to controls. These heterogenous findings indicate that ADG is also dependent on other factors such as genetics, husbandry system and feed quality. In our case, we can only hypothesize that feeding and husbandry conditions were already adequate to allow for a high average daily weight gain (around 500 g/day for rearing and 750 g/day during fattening for both treatment groups). We included meat pH because we hypothesized that FHE-fed pigs might have been less susceptible to stressors such as transport, unloading and lairage time at the abattoir. A better stress resilience might have led to a higher meat pH (69). A lack of effect could be explained by the fact that all study pigs were used to changing environmental conditions as they lived in improved husbandry systems with outdoor runs and more exposure to outdoor stimuli compared to pigs only kept indoors. This can also be confirmed by results of a study comparing improved husbandry conditions of the same label to fully conventionally kept pigs, where the prevalence of pigs with less than pH of 6 was lower in label pigs compared to conventionally kept pigs (61).

## 5 Conclusions

Since only few indicators showed differences between treatment groups, results should be interpreted with care, given the limitations of an on-farm study and the limited observation periods. Furthermore, since FHE contained probiotics and phytobiotics, their effects cannot be distinguished. Our findings indicate that the FHE's probiotic and/or phytotherapeutic properties may have potential to enhance aspects of welfare, namely reduced prevalence of rearing pigs with tail tissue loss and lower incidence of coughing in fatteners. In light of the ongoing societal and political discussions within the EU regarding intact tails and the increasing regulatory pressure to avoid tail docking, FHE may serve as one element in a multifactorial strategy to reduce tail biting in rearing and fattening pig systems. Furthermore, our findings could indicate that FHE may also positively influence the occurrence of coughing during the fattening period. This could not only be relevant for conventional housing systems, but also within welfare-oriented housing systems with straw bedding, where high dust levels pose a significant risk to respiratory health. FHE may thus offer additional benefits for promoting pig health and welfare, potentially reducing antibiotic use. While these findings are promising, they warrant further investigation and confirmation.

## Data Availability

The raw data supporting the conclusions of this article will be made available by the authors, without undue reservation.
