# Supplementary material for: Do fermented herbal extracts affect pig behavior, health and productivity? An on-farm study
Source: Front Vet Sci. 2026 Jun 4;13:1812716. doi: 10.3389/fvets.2026.1812716 (PMC13275253; doi:10.3389/fvets.2026.1812716)
Supplement: Supplementary file 1 [file Data_Sheet_1.docx]

Supplementary Material

The right gut feeling? Effects of fermented herbal extracts on pig behavior, health and productivity

Natalia Nöllenburg^1*^, Barbara Metzler-Zebeli^2^, Katharina Schobersberger^1^, Christoph Winckler^1^, Christine Leeb^1^

^1^Institute of Livestock Sciences, Department of Agricultural Sciences, BOKU University, 1180 Vienna, Austria

^2^Unit Nutritional Physiology and Functional Plant Compounds, Clinical Department for Farm Animals and Food System Transformation, University of Veterinary Medicine Vienna, 1210 Vienna, Austria

*** Correspondence:**natalia.noellenburg@boku.ac.at

Keywords: animal welfare, rearing, fattening, microbiota-gut-brain-axis, tail length

**Supplementary Table 1.** Ingredient and chemical composition of starter and fattening diets fed at the time point of fecal collections.

|  | Farm A |  |  |  | Farm B |  |  |
| --- | --- | --- | --- | --- | --- | --- | --- |
|  | Ingredient (%) | Rearing  diet | Fattening diet |  | Ingredient (%) | Rearing  diet | Fattening diet |
|  | Corn-Cobb-Mix | 44.0 | 50.0 |  | Barley | 24.4 | 54.0 |
|  | Barley | 16.8 | 12.5 |  | Corn | 26.2 | 25.0 |
|  | Wheat | 5.6 | 10.0 |  | Wheat | 22.9 | - |
|  | Oat | 2.8 | 2.5 |  | Soybean meal 44 | 19.9 | 18.0 |
|  | Corn | 2.8 | 0 |  | Schaumann Fiber concentrate^3^ | 2.5 | - |
|  | Solan 135A GVO-free^2^ | 28.0 | 25.0 |  | Schaumaphos F-VM 90 ATG^3^ | 4.2 | - |
|  |  |  |  |  | Schaumaphos M 70/4000^3^ | - | 3.0 |
|  |  |  |  |  |  |  |  |
|  | Chemical composition |  |  |  | Chemical composition |  |  |
|  | Dry matter (g/kg) | 804 | 768 |  | Dry matter (g/kg) | 891.5 | 755 |
|  | g/kg (dry matter basis) |  |  |  | g/kg (dry matter basis) |  |  |
|  | Crude protein | 179 | 175 |  | Crude protein | 178 | 180 |
|  | Crude fiber | 39 | 42.5 |  | Crude fiber | 60.0 | 52.5 |
|  | Ether extract | 35.5 | 38.0 |  | Ether extract | 31.0 | 30.5 |
|  | Crude ash | 55.5 | 47.5 |  | Crude ash | 55.5 | 54.5 |
|  | Nitrogen-free extracts | 691 | 698 |  | Nitrogen-free extracts | 675 | 682 |
|  | Starch | 528 | 526 |  | Starch | 513 | 515 |
|  | Sugar | 35.0 | 35.5 |  | Sugar | 42.5 | 37.5 |
|  | Metabolizable energy, MJ ME/kg (dry matter) | 15.18 | 15.21 |  | Metabolizable energy, MJ ME/kg (dry matter) | 14.33 | 14.57 |

DM, dry matter; ingredient and chemical composition of the basal feed mixture was identical for the FHE and CON groups; the only difference was FHE supplementation of 1% (on DM basis).

^1^Edlinger GmbH, Aschbach, Austria.

^2^Solan, SOLAN Kraftfutterwerk Schmalwieser GesmbH & Co KG, Bachmanning, Austria.

^3^H.Wilhelm SCHAUMANN GmbH & Co KG, Brunn am Gebirge, Austria.

**Supplementary Table 2.** Ethogram of different types of play behavior (complementing table 2)

| **Behavior** | **Definition** | **Source** |
| --- | --- | --- |
| Pivot | Jump on spot to face in a different direction  (rotation of at least 90°) | Newberry et al. 1988  Donaldson et al. 2002 |
| Hop | Jump up and down on the spot while facing in one direction | Newberry et al. 1988 |
| Scamper | Sequence of two or more forward hops in rapid succession | Newberry et al. 1988  Donaldson et al. 2002 |
| Gambol | Running across the pen, occasionally accompanied by nudging pen mate gently | Bolhuis et al. 2005 |
| Romp | Combination of mutual pushing and gentle fighting, often accompanied by chasing | Zonderland et al. 2011 |

**Supplementary Table 3.** Medical treatments during rearing: Mean incidence of antibiotic treatments (per 100 animals per rearing phase) of CON (n=12) and FHE (n=12) pens

| **Farm** | **A** | | | | | | **B** | | | | | |
| --- | --- | --- | --- | --- | --- | --- | --- | --- | --- | --- | --- | --- |
| **Batch** | **1** | | **2** | | **3** | | **1** | | **2** | | **3** | |
| **Treatment** | CON | FHE | CON | FHE | CON | FHE | CON | FHE | CON | FHE | CON | FHE |
| **Diarrhea** |  |  | 100 | 100 |  |  |  |  |  |  |  |  |
| **Lameness** |  |  |  |  |  | 2.4 |  |  |  |  |  |  |
| **Tail injuries** |  |  |  |  |  |  |  |  | 100 |  | 100 |  |
| **Other** (e.g. weakness, inappetence) |  | 6 |  |  |  |  |  | 2.5 | 2.5 |  |  |  |

**Supplementary Table 4.** Medical treatments during fattening: Mean incidence of antibiotic treatments (per 100 animals per fattening phase) of CON (n=9) and FHE (n=9) pens

| **Farm** | **A** | | | | | | **B** | | | | | |
| --- | --- | --- | --- | --- | --- | --- | --- | --- | --- | --- | --- | --- |
| **Batch** | **1** | | **2** | | **3** | | **1** | | **2** | | **3** | |
| **Treatment** | CON | FHE | CON | FHE | CON | FHE | CON | FHE | CON | FHE | CON | FHE |
| **Respiratory disease** |  |  |  |  |  |  | 10 | 32.5 | 5 | 5 | 5 |  |
| **Lameness** |  |  |  | 6 |  | 2.5 | 22.5 | 5 |  | 7.5 | 5 |  |
| **Other** (e.g. weakness, inappetence) |  |  |  |  |  |  | 5 |  |  |  |  |  |

**Supplementary Table 5.** Excluded pens for the indicator “tail shorter” during the fattening phase

| **Batch** | **Visit*** | **Farm A** | **Farm B** |
| --- | --- | --- | --- |
| 1 | 1 | – | Entire batch |
|  | 2 | – | Entire batch |
|  | 3 | – | Entire batch |
| 2 | 1 | – | – |
|  | 2 | – | – |
|  | 3 | – | – |
| 3 | 1 | – | – |
|  | 2 | – | 1 FHE pen |
|  | 3 | 1 CON pen | 1 FHE pen; 2 CON pens |

*Visit 1 = end of rearing, visit 2 = middle of fattening, visit 3 = end of fattening
